# Discrepancies between workers with disabilities and their supervisors in reported work accommodations and associations with return to work

**DOI:** 10.1186/s12889-023-15038-7

**Published:** 2023-03-18

**Authors:** Joke Jansen, Nicole Snippen, Pierre Koning, Cécile Boot, Raun van Ooijen, Sandra Brouwer

**Affiliations:** 1 Department of Health Sciences, Community and Occupational Medicine, University of Groningen, University Medical Center Groningen, PO Box 196, Groningen, 9700 AD The Netherlands; 2 Department of Economics, Vrije Universiteit Amsterdam, Amsterdam, The Netherlands; 3 Department of Public and Occupational Health, Amsterdam UMC location Vrije Universiteit Amsterdam, Amsterdam, The Netherlands; 4 Societal participation & Health, Amsterdam Public Health research institute, Amsterdam, The Netherlands

**Keywords:** Return to work, People with disabilities, Longitudinal survey

## Abstract

**Background:**

The aims of this study were: (1) to explore the frequency of discrepancies in work accommodations reported by workers and their supervisors, and (2) to investigate whether these discrepancies are associated with full return to work (RTW).

**Methods:**

We used data from a longitudinal survey study of long-term sick-listed workers and their supervisors (*n* = 406). Discrepancies in reports on implementing eight types of work accommodations were explored. Logistic regression analyses were conducted to test associations between discrepancies in reported work accommodations and odds of full RTW 27 months after the sick-leave onset.

**Results:**

Discrepancies were the lowest for the work accommodation therapeutic RTW (53%) and the highest (85%) for job training or education and reimbursement of therapy or treatment. Four out of eight types of work accommodations were more often reported by workers than by their supervisors. Only a discrepancy on a job reassignment within the organization was associated with lower odds of full RTW (OR 0.56, 95%-CI 0.36–0.88).

**Conclusion:**

We found substantial discrepancies in the reported implementation of work accommodations between workers and their supervisors. Future research should focus on disentangling mechanisms that lead to discrepancies to avoid inefficiencies in the RTW process.

## Background

Work accommodations, such as job task modifications, workplace adjustments, or reduced working hours, play an essential role in enabling long-term sick-listed workers to return to work (RTW), either fully or partially [1, 2]. To foster the implementation of work accommodations, many countries have developed long-term sick leave policies entitling long-term sick-listed workers to work accommodations to enable them to resume work [3]. Usually, long-term sick-listed workers and their supervisors have a shared responsibility in deciding on the type of suitable work accommodations [1]. They are typically expected to collaborate in the RTW process, but each has a distinct role and responsibilities [4]. While supervisors are primarily responsible for initiating and implementing work accommodations by modifying the terms and conditions of employment or facilitating adjustments in the workplace [5], workers are expected to collaborate and communicate their needs to their supervisors.

Since workers and supervisors have a shared responsibility in implementing work accommodations, one might expect that workers and supervisors would report similarly on which work accommodations have been implemented during the worker’s RTW process. Stated differently, any discrepancies in perceived accommodations may point to inefficiencies in the RTW process. Indeed, prior studies have found such discrepancies in the perceptions of workers and supervisors about work functioning, supervisory skills, and safety climate. These studies show that these discrepancies are negatively associated with work-related outcomes like job satisfaction or organizational commitment [6–9]. Although this evidence highlights the potential importance of discrepancies in perceptions of workers and supervisors for several work-related outcomes, no prior research has investigated the possible association between discrepancies in reported implementation of work accommodations and RTW after long-term sick leave.

So far, the literature on work accommodations has provided evidence that there are discrepancies between workers and supervisors in reported reasons for accommodations not being fully granted, as well as between implemented and desired work adjustments [10, 11]. For discrepancies in the reported implementation of work accommodations, such evidence is lacking. Arguably, these discrepancies in how workers and supervisors perceive the implementation of work accommodations may have significant consequences for the RTW process. Consequently, considering the perspectives of both supervisors and workers may provide a more complete picture of adequate implementation of work accommodations by supervisors [9, 12].

This study, therefore, aimed to (i) explore the frequency of discrepancies in reported work accommodations between workers that have been sick-listed for longer than 9 months and their supervisors and (ii) investigate whether these discrepancies are associated with the odds of full RTW of these workers (i.e., working the same hours as before reporting sick at the same or another employer).

## Materials and methods

### Design

**Fig. 1:**
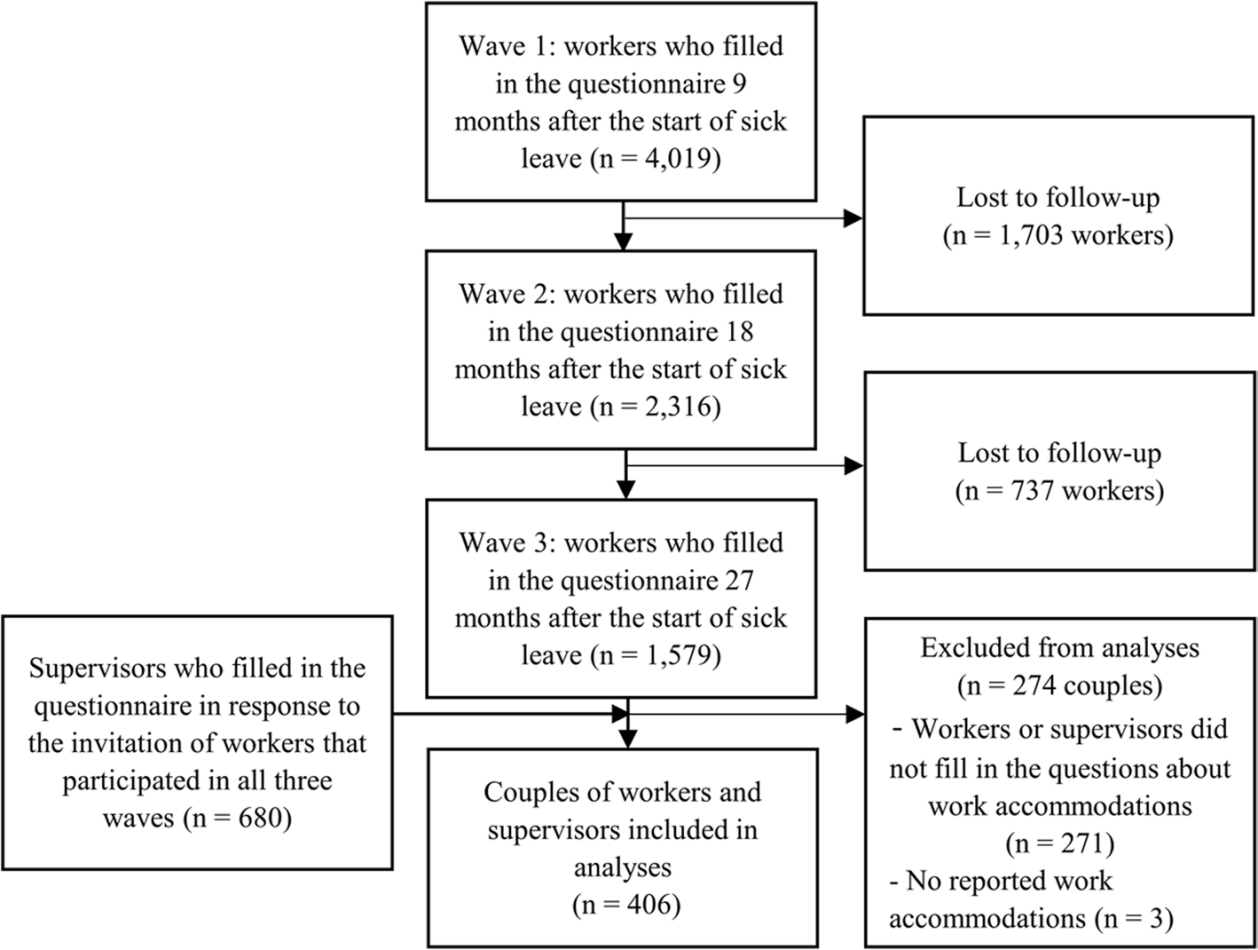
A flow-chart depicting the inclusion and exclusion of workers and supervisors in the questionnaire waves and the included couples in the analyses

We conducted a secondary analysis on data of a longitudinal survey entitled “pathways-to-disability-survey” in the Netherlands in 2007 [13]. This survey was conducted among 4,019 long-term sick-listed workers that had been sick-listed for more than 9 months. These workers were reported sick within three weeks before or after January 1, 2007, and had not (fully) returned to work nine months later. The data collection among workers consisted of three waves. Workers were asked to fill in a questionnaire at 9 months, 18 months, and 27 months after starting sick leave. The data collection among supervisors consisted of one wave (> 27 months after the start of the worker’s sick leave). At 27 months after the start of sick leave, workers who filled in all three questionnaires (*n* = 1,579) were requested to ask their supervisors to participate in the survey. In total, 680 supervisors filled in the questionnaire in response to the invitation from participating workers. In this study, we only used data from complete cases, in the sense that we only included couples in which both the worker and supervisor responded to the questions about work accommodations. Furthermore, we included only couples in which at least one person indicated that one or more work accommodations were implemented. With these restrictions, the final study sample consisted of 406 couples of workers and supervisors. There were no statistically significant differences between included and excluded couples with regard to baseline characteristics (i.e., gender, age, educational level, type of disability) and RTW outcomes of workers. The sample selection process is visualized in Fig. 1.

### Measures

#### Primary outcome

The primary outcome measure was full RTW (i.e., working the same hours as before reporting sick) at the same or another employer at 27 months after starting sick leave, measured in the third wave. Workers were asked to indicate their RTW status using the following response options: no RTW, partial RTW, and full RTW. We recoded the variable into a binary variable: full RTW versus partial or no RTW.

#### Implementation of work accommodations

In this study, we used data of workers and their supervisors about the implementation of work accommodations undertaken by the supervisor. Reports about the work accommodations implemented by the supervisor were measured using a multiple response item. In this item, workers and supervisors were asked to indicate which work accommodations were implemented by the supervisor to support the worker to return to work or to continue employment. Workers and supervisors could select one or more answers from the following categories: (1) reimbursement of therapy or treatment, (2) counseling or coaching, (3) job reassignment within the organization, (4) therapeutic RTW: modified job duties recognizing work as therapeutic in itself, (5) workplace adaptation or equipment, (6) job training or education, (7) reduction in working hours, and (8) task modifications. For workers, we considered a work accommodation to be reported when the worker had selected this type of work accommodation in at least one of the three waves.

To explore the frequency of discrepancies, couples were grouped based on combined worker-supervisor responses on implementing specific work accommodations. For this first research question, for each work accommodation we only looked at couples in which at least one person had reported the implementation of the work accommodation. For each of the eight work accommodations, couples were grouped into one of the three following categories: (i) only reported by the worker, (ii) reported by workers and supervisors, and (iii) only reported by the supervisor.

For the second research question, to investigate whether discrepancies in reported work accommodations are associated with RTW, binary variables were created for the eight work accommodations. For these binary variables, couples were grouped based on whether both persons in a couple reported that a work accommodation had or had not been implemented (i.e., the agreement group) or whether only one person in the couple had reported the implementation of the work accommodation (i.e., the discrepancy group).

#### Sociodemographic measures

At baseline, data was collected about the following worker characteristics: age in years (categorized as < 34, 35–44, 45–54, 55–65), gender (male/female), educational level (low/medium/high), and type of disability (somatic/mental/mixed) (see Table 1). No data was available on the sociodemographic characteristics of participating supervisors because this was not collected in the questionnaires.

### Analyses

Quantitative data were analyzed using SPSS version 26. Descriptive statistics (e.g., frequencies and percentages) were used to describe the study sample and explore discrepancies between workers and supervisors about implemented work accommodations. We performed Chi-square analyses and logistic regression analyses to investigate whether discrepancies in reported work accommodations are associated with full RTW, applying a significance level of 0.05. In the logistic regression analyses, age, gender, and disability type (somatic/mental/mixed) were included as covariates.

## Results

As explained, 406 workers and supervisors were included. A slight majority of workers was female (52.6%). Most workers were between 45 and 65 years old (73.2%) and had received a medium or high level of education (63.6%). Most workers (71.6%) had a somatic disease, particularly musculoskeletal disorders (37.2%) and 61.3% of the workers reported full RTW at 27 months after starting sick leave. Of the workers that had not fully returned to work at 27 months (38.7%), 40.8% had partially returned to work. More detailed demographic information of participating workers is provided in Table 1.

**Table 1:** Workers’ characteristics

Characteristics	Total sample (*N* = 406)	
Age in categories (years)		
< 34	29	(7.1%)
35–44	80	(19.7%)
45–54	166	(40.9%)
55–65	131	(32.3%)
Gender		
Male	192	(47.3%)
Female	214	(52.7%)
Disability type		
Somatic	292	(71.7%)
Mental	81	(20.0%)
Mixed	34	(8.4%)
Educational level^a^		
Low	138	(34.0%)
Medium	129	(31.8%)
High	129	(31.8%)
Full RTW at 27 months after the start of sick leave		
Yes	249	(61.3%)
No	157	(38.7%)

^a^Low educational level = primary education, pre-vocational secondary education (VMBO); Medium educational level = senior general secondary education (HAVO), pre-university education (VWO), secondary vocational education (MBO); High educational level = higher professional education (HBO), university education (WO), doctorate (PhD). Contains *n* =10 missing observations

### Discrepancies in reported work accommodations between workers and supervisors

**Fig. 2:**
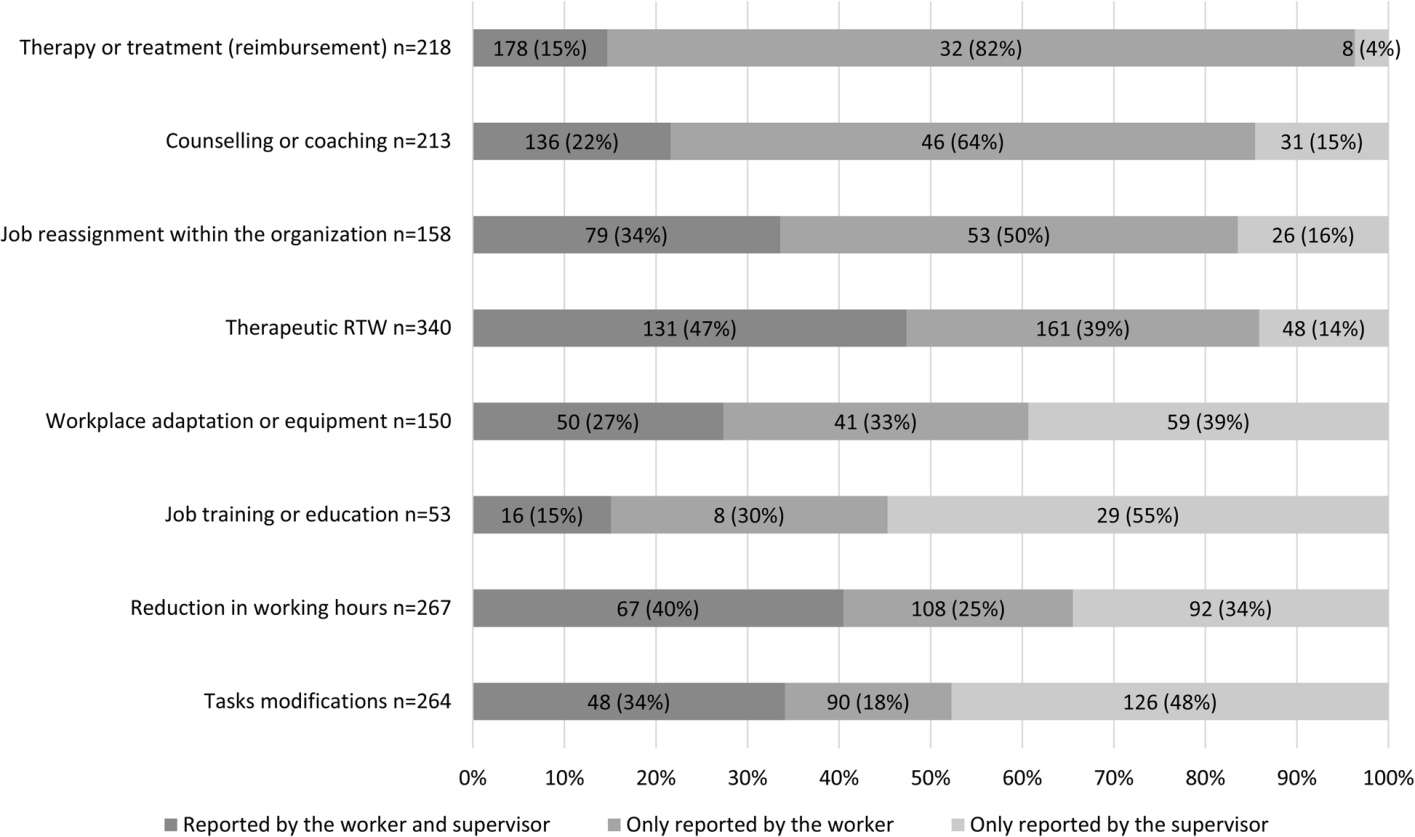
Implemented work accommodations (n) reported by only the worker, the worker and the supervisor and only the supervisor

The number and percentages of discrepancies between workers and supervisors on the reported work accommodations are presented in Fig. 2. Within the couples in which an accommodation was reported by at least one person, the discrepancies ranged between 53 and 85%. The lowest discrepancy was found for therapeutic RTW, the highest (85%) for job training or education and reimbursement of therapy or treatment. Workers reported four work accommodations more often than their supervisors: reimbursement of therapy or treatment (82%), counseling or coaching (64%), job reassignment within the organization (50%) and therapeutic RTW (39%). For instance, regarding the work accommodation therapy or treatment, the implementation was in most cases only reported by the worker (82%). Similarly, supervisors reported four work accommodations more often than the worker: workplace adaptation or equipment (39%), job training or education (55%), reduction in working hours (34%) and tasks modifications (48%).

### Associations between discrepancies in reported work accommodations and RTW

Chi-Square Tests of Independence were performed to assess the relationship between RTW status at 27 months after the first day of sick leave and the eight work accommodations (Table 2). The results showed that the proportion of workers that had fully returned to work differed depending on whether or not there was a discrepancy in the reported implementation of a job reassignment within the organization (*χ*^*2*^ = 5.85; *df* = 1; *p* = .02). Workers who agreed with their supervisor about whether or not there had been a job reassignment within the organization were more likely to be fully back at work at 27 months. No other statistically significant associations were found.

Consistent with the Chi-square tests, logistic regression analysis showed that a discrepancy in the reported implementation of a job reassignment within the organization was associated with lower odds for RTW (OR 0.56, 95%CI: 0.36–0.88). No other statistically significant associations were found. The associations are presented in Table 3.

**Table 2:** Crosstabs and Chi-square Tests of Independence to determine the associations of discrepancies with reported work accommodations

Work accommodation	Full RTW at 27 months (n, % within row)		Chi square test (1, *N* = 406)	
	No	Yes	χ^2^	*p*
Therapy or treatment (reimbursement)			0.40	0.53
Agreement	82 (37.3%)	138 (62.7%)		
Discrepancy	75 (40.3%)	111 (59.7%)		
Counseling or coaching			2.46	0.12
Agreement	100 (41.8%)	139 (58.2%)		
Discrepancy	57 (34.1%)	110 (65.9%)		
Job reassignment within organization			5.85	0.02*
Agreement	106 (35.2%)	195 (64.8%)		
Discrepancy	51 (48.6%)	54 (51.4%)		
Therapeutic RTW			2.85	0.09
Agreement	96 (42.3%)	131 (57.7%)		
Discrepancy	61 (34.1%)	118 (65.9%)		
Workplace adaptation or equipment			0.04	0.85
Agreement	114 (38.4%)	183 (61.6%)		
Discrepancy	43 (39.4%)	66 (60.6%)		
Job training or education			0.04	0.85
Agreement	139 (38.5%)	222 (61.5%)		
Discrepancy	18 (40.0%)	27 (60.0%)		
Reduction in working hours			0.27	0.60
Agreement	98 (39.7%)	149 (60.3%)		
Discrepancy	59 (37.1%)	100 (62.9%)		
Task modifications			1.18	0.28
Agreement	95 (40.9%)	137 (59.1%)		
Discrepancy	62 (35.6%)	112 (64.4%)		

*p < 0.05

**Table 3:** Logistic regression of discrepancies about work accommodations being implemented by the supervisor associated with odds of actual RTW of workers on long-term sick leave

Discrepancy on work accommodations (binary)^a^ (agreement = ref)	Odds ratio	Lower 95%CI	Upper 95%CI	*P*-value*
Therapy or treatment (reimbursement)	1.42	0.94	2.14	0.72
Counseling or coaching	1.35	0.89	2.06	0.16
Job reassignment within organization	**0.56**	**0.36**	**0.88**	0.01*
Therapeutic RTW	1.42	0.94	2.14	0.09
Workplace adaptation or equipment	0.96	0.60	1.52	0.85
Job training or education	0.95	0.50	1.80	0.87
Reduction in working hours	1.11	0.73	1.70	0.64
Task modifications	1.22	0.81	1.83	0.35

^a^Adjusted for age, gender, and type of disability; * *p* < .05

## Discussion

Our findings on discrepancies in reported work accommodations, in case an accommodation was reported by at least one person (Fig. 2), show substantial discrepancies between workers and their supervisors in their reports on implemented work accommodations. Within the couples in which an accommodation was reported by at least one person, discrepancies in reported implementation of the eight work accommodations were the lowest for therapeutic RTW (53%) and the highest (85%) for job training or education and reimbursement of therapy or treatment. Reimbursement of therapy or treatment, counseling or coaching, job reassignment within the organization, and therapeutic RTW were more often reported by workers than their supervisors. Notably, the present study indicates that a discrepancy between workers and supervisors on whether a job reassignment within the organization was implemented was associated with a 50%-point lower probability of full return to work at 27 months after the start of sick leave. Other than for job reassignment, no statistically significant associations were found between discrepancies in reported work accommodations and full RTW.

The observed differences in discrepancies between the eight work accommodations may well reflect different levels of involvement of the worker or the supervisor in implementing each work accommodation. For example, reimbursement of therapy and coaching was more often only reported by the worker, while a reduction in working hours and task modifications were more often only reported by the supervisor. A possible explanation is that the work accommodations that the worker more often reports are initiated by other stakeholders arranging the accommodation, like an outplacement agency or a case manager. In these cases, the supervisor is not necessarily informed, for instance, because of privacy regulations. On the other hand, supervisors more often reported the implementation of work accommodations that have a direct and formal impact on the job, like reduction of working hours and modifications in work tasks. This might be explained by different perceptions of how these work accommodations are defined. Workers and supervisors thus have both overlapping and distinct roles in the RTW process within their shared responsibility. The overlapping roles relate to being actively involved in the RTW process. The distinct roles relate to the specific tasks workers and supervisors have regarding implementing work accommodations. While the worker usually collaborates with work accommodations, the supervisor’s primary role is to ensure that the work accommodations are implemented [14]. When workers and supervisors do not commit to a shared responsibility, their distinct roles may (in part) explain the discrepancies found regarding some work accommodations [4].

Except for one type of work accommodation (i.e., job reassignment within the same organization), discrepancies in reported work accommodations were not associated with full RTW. Although there are only minor indications that discrepancies are associated with full RTW, future research should focus on disentangling mechanisms that lead to discrepancies, as discrepancies may lead to inefficiencies in the RTW process that should be avoided. The only significant finding concerned the perceived occurrence of job reassignments within the organization. Job reassignment within the same organization is one of the most commonly implemented work accommodations, along with reductions in working hours and task modifications [15, 16]. The start of a new job position or having new tasks within the same organization is usually implemented when other measures are not feasible and might be an intervention of last resort [15]. This in itself may indicate that full RTW is more difficult, which is also mirrored by fewer hours worked by long-term sick-listed workers and workers with disabilities [16] and lower residual employment durations after work resumption [17]. In addition, workers and supervisors may have different perceptions of whether the specific work accommodations were implemented. This highlights that empirical analyses regarding implemented work accommodations are largely contextual and include measurement errors. However, our results do not indicate that potential discrepancies that could result from misreporting if workers are dissatisfied with the RTW process are associated with worse reported RTW outcomes. At the same time, other measures that proxy perceptions regarding the RTW process, such as the feeling of injustice or dissatisfaction with work, are probably more likely to be significantly associated with perceptions regarding the RTW process.

### Strengths and limitations

A strength of this study was the use of data from couples of workers and their supervisors. Previous studies examining associations between work accommodations and RTW were based on information from the worker or the supervisor perspective, but not from both. By comparing information from both the worker and the supervisor, we were able to show that discrepancies are prevalent, and that a combined investigation of workers’ and supervisors’ perspectives provides different, likely more complete, information on the implementation of work accommodations. Another strength of this research is the long-term follow-up of the survey study, which allowed us to investigate the associations of discrepancies in reported work accommodations with full RTW at 27 months after the start of sick leave.

This study also has some limitations. While we performed secondary analyses on data from a survey conducted in 2007, we assume that the discrepancies still exist nowadays because no major institutional changes were made since then affecting the RTW process. Furthermore, we cannot rule out information bias because workers and supervisors completed the questionnaires at different points in time. The recall period regarding the implemented work accommodations differed substantially between supervisors (> 27 months after the start of sick leave) and workers (at 9 months, 18 months, and 27 months after the start of sick leave), which may have affected our findings. Possibly, the timing of the supervisor questionnaire led to a larger risk of recall errors by supervisors on the implementation of work accommodations. This could thus explain some of the discrepancies between workers and supervisors in reported work accommodations. In addition, no detailed information on the supervisor was collected in the survey; therefore, we could not control for the supervisor-characteristics.

### Implications

When implementing work accommodations during long-term sick leave, workers and their supervisors have a shared responsibility for the success of the RTW process. Meaning that workers and their supervisors collaboratively make decisions and discuss the options and the likely benefits and harms of each option while considering the worker’s values, preferences, and circumstances. However, from our analysis, we infer that there are substantial discrepancies in reported work accommodations between workers and their supervisors. Although the analyses did not show significant results concerning the effect of discrepancies in seven work accommodations on RTW, we cannot exclude the possibility that proxies for the actual collaboration of supervisors and workers are still important. For example, in the Netherlands, workers and supervisors write a RTW plan 12 weeks after the onset of sick leave. Although this action is required by law, it is possible that workers and supervisors only have a formal conversation about the RTW plan without making specific agreements about the implementation of work accommodations and who is responsible for the implementation process [18]. Even though this study is a secondary analysis of data collected with another purpose, the data show the presence of large discrepancies that deserve further exploration in future studies, as these discrepancies may be a barrier for successful RTW. Future research should therefore focus on disentangling mechanisms that lead to discrepancies in reported work accommodations, to avoid inefficiencies in the RTW process. This requires insight into aspects of shared decision-making, and in the communication between supervisors and workers about the implementation of work accommodations.

## Conclusion

We found substantial discrepancies in the reported implementation of work accommodations between workers and their supervisors. In case an accommodation was reported by at least one person, workers more often reported work accommodations like coaching and reimbursement for therapy than their supervisors. In contrast, supervisors more often reported work accommodations like task modification and working hours reduction than workers. Except for one type of work accommodation, i.e. job reassignment within the same organization, discrepancies in reported work accommodations were not associated with full RTW. Future research should focus on disentangling mechanisms that lead to discrepancies in reported work accommodations to avoid inefficiencies in the RTW process.

## Data Availability

The data that support the findings of this study are available from the Dutch Social Security Institute but restrictions apply to the availability of these data, which were used under license for the current study, and so are not publicly available. Data are however available from the author Sandra Brouwer, sandra.brouwer@umcg.nl, upon reasonable request and with permission of the Dutch Social Security Institute.
